# COVID consequences continue

**DOI:** 10.1111/iwj.14113

**Published:** 2023-02-14

**Authors:** Douglas Queen

As healthcare practitioners and perhaps human beings, we are all too familiar with the long‐term effects of the COVID pandemic. Your first thought might be the so‐called ‘long COVID’.

Most people who suffer from COVID‐19 recover within a few weeks, some however might have symptoms that are long lasting post infection.^1^ This is a select few unfortunate individuals, but for the rest of us, it's not plain sailing either.

Mid‐pandemic the United Nations stated, ‘We are facing a global health crisis unlike any in the 75‐year history of the United Nations—one that is killing people, spreading human suffering, and upending people's lives. But this is much more than a health crisis. It is a human, economic, and social crisis’.^2^

Measures implemented to slow the spread of COVID‐19 and prevent severe outcomes and deaths also affected our physical, mental, emotional, and spiritual wellness, the health care system, environment, and economy.^3^ In 2023 as we see the pandemic continuing to be a threat to our health, albeit a more understood disease, COVID‐19 continues to impact our everyday lives in many ways. That is its impact on our economy, work lives, social lives, and health in different but impactful ways.

The surge of influenza and respiratory syncytial virus (RSV) infections are currently overwhelming hospitals and their intensive care units raising bed occupancy to over 80% in many regions.^4^ As RSV subsides, the winter COVID‐19 surge takes over. Infections are not the only reason patients require hospital care. Other winter‐related injuries requiring hospital care include falls, heart attacks, hypothermia, and carbon monoxide poisoning.^4^ The over‐stretched healthcare system continues.

Some current challenges that impact health care in different ways include:

## 1 INFLATION IS A THREAT TO OUR ECONOMY AND OUR HEALTH

Inflation exposed the weaknesses of our fragile economy.^5^ Food prices are significantly higher than a year ago while wages continue to grow slowly. These economic challenges for most weaken their position regarding paying for healthcare, whether that is therapeutic approaches (e.g., wound care supplies or COVID vaccines) or diagnostics (e.g., COVID tests). These economic impacts will be costly to our healthcare system. The ill will become sicker, miss work and may require hospitalisation, providing the perfect circular storm.

## 2 SUPPLY CHAINS HAVE NOT RECOVERED

The inability to purchase a new car or major appliance was seen as inconvenient but compared with difficulty accessing basic goods, including certain medications it was a minor inconvenience.^6^ Our supply chain models were designed around ‘Just‐in‐Time’ and work well when they are reliable, but most are not flexible enough to efficiently deal with significant disruptions.

Eggs, toilet paper and fresh produce rely on a fragile system of distribution. Basic medicines and therapeutics are also vulnerable to such disruptions. Supply chain disruption has already caused a shortage of common medications like antibiotics and cough medications. Disruptions increase the price of medications creating problems for those who pay, whether these are individuals or healthcare systems.

## 3 HEALTHCARE DELIVERY

Concerns about contracting COVID‐19 and the response measures we lived with to reduce transmission have impacted our health system and how we seek care moving forward.^7^ The COVID‐19 pandemic has changed the way patients access health care and how health care is delivered using virtual options, even in this post‐pandemic period.^7^

For example^7^:

- During the pandemic non‐urgent surgeries were postponed or not scheduled at all, to limit the spread of COVID‐19, and to ensure that hospitals had the capacity to address COVID patient needs. This has changed somewhat post‐pandemic, but the backlog now needs to be addressed. Long wait times for non‐urgent surgeries can result in poorer health outcomes including mortality, quality of life, and quality of care, as well as take a psychological toll on patients and families, including anxiety and distress. So how do we address the backlog and improve the healthcare we deliver?
- Increases in wait times for diagnostic services can result in downstream delays in health care service access, including diagnosis for surgical referral, biopsy, and post‐surgical rehabilitation care.
- During the COVID‐19 pandemic, the way we access health care has evolved to include more virtual options. Virtual health care delivery has the benefit of reaching more people, particularly in rural and remote communities where they generally had a shortage of in‐person health services even before the pandemic. However, balance that with the workforce shortage of today, and these pandemic benefits may not be restricted to rural and remote communities (i.e., virtual options are here to stay).
- Simultaneously, health care workers working in and who continue to work under more intense conditions during and post‐pandemic continue to burn out and workforce shortages are a consequence. Every aspect of care delivery is experiencing this impact and most geographies around the world are having to plan care accordingly.
- The COVID‐19 pandemic and response measures posed challenges for some individuals to access primary care (family doctors/general practitioners, nurse practitioners). Increased isolation because of the lockdown and fear of catching COVID‐19 has caused some individuals to only seek care when their condition worsens.
- Access to speciality services and clinics was impacted by COVID response measures and continues to be an issue. This leads to longer wait periods between receiving a referral and consultation, in addition to delays in treatment (e.g., cancer, asthma, wound care).

## 4 CONCLUSION: WE CAN PAY NOW OR PAY DEARLY LATER

The impact on and erosion of our global health systems have never been greater.^8^ Yet healthcare payers, including governments, remain cautious with healthcare spending.^8^ This funding environment existed prior to the pandemic, disappeared to a degree during the pandemic, but surely should not come back post‐pandemic. Investment now will reduce the future more significant investment to clean up the mess caused by COVID‐19.^8^ It's time to recreate our healthcare systems to meet the needs of today's population. Let us learn from the pandemic and not continue to suffer because of the pandemic. This includes wound care!
